# Evaluating Small Intestinal Motility in a Rat Model of Adolescent Irritable Bowel Syndrome

**DOI:** 10.14789/jmj.JMJ21-0050-OA

**Published:** 2022-06-09

**Authors:** MASAMICHI SATO, TAKAHIRO KUDO, NOBUYASU ARAI, REIKO KYODO, KENJI HOSOI, KEITA SAKAGUCHI, TAMAKI IKUSE, KEISUKE JIMBO, YOSHIKAZU OHTSUKA, TOSHIAKI SHIMIZU

**Affiliations:** 1 Department of Pediatrics and Adolescent Medicine, Juntendo University Graduate School of Medicine, Tokyo, Japan; 1 Department of Pediatrics and Adolescent Medicine, Juntendo University Graduate School of Medicine, Tokyo, Japan

**Keywords:** adolescent, enterochromaffin cells, gastrointestinal motility, irritable bowel syndrome, serotonin 3 receptor

## Abstract

**Objectives:**

The correlation between altered small intestinal motility and irritable bowel syndrome is not well evaluated. This study aimed to assess the small intestinal and colonic transits in an adolescent irritable bowel syndrome rat model with restraint stress and determine the role of small intestinal motility in the irritable bowel syndrome pathophysiology.

**Materials:**

Restraint stress was utilized to prepare adolescent irritable bowel syndrome rat models that were evaluated for clinical signs, including stool frequency and diarrhea. The small intestinal motility and transit rate were also evaluated.

**Methods:**

The amounts of mRNA encoding corticotropin-releasing hormone, mast cell, and serotonin (5-Hydroxytryptamine) receptor 3a were quantified using real-time polymerase chain reaction; the 5-Hydroxytryptamine expression was evaluated using immunostaining.

**Results:**

Restraint stress significantly increased the number of fecal pellet outputs, stool water content, and small intestinal motility in the adolescent irritable bowel syndrome rat models. There was no difference in real-time polymerase chain reaction results; however, immunostaining analysis revealed that 5-Hydroxytryptamine expression in the small intestine was significantly increased in the adolescent irritable bowel syndrome rat models.

**Conclusions:**

In the rat model of adolescent irritable bowel syndrome with restraint stress, we observed an increase in small intestinal and colonic motility. In the small intestine, enhanced 5-Hydroxytryptamine secretion in the distal portion may be involved in increasing the small intestinal motility. Although the present study focused on 5-Hydroxytryptamine, further investigation of other factors that regulate intestinal peristalsis may lead to the establishment of more effective treatment methods for adolescent irritable bowel syndrome.

## Introduction

Irritable bowel syndrome (IBS) is a common functional gastrointestinal disorder characterized by the symptoms of abdominal pain and altered bowel habits, such as diarrhea or constipation, in the absence of any organic diseases. Although the pathophysiology of this disease remains uncertain, sufficient compelling evidence has accumulated to indicate that IBS is a multifactorial syndrome resulting from interactions among gastrointestinal motility, visceral hypersensitivity, intestinal inflammation, gastrointestinal infection, fecal flora alterations, bacterial overgrowth, food sensitivity, genetic factors, and psychosocial dysfunction^1)^. The prevalence of IBS is high in children, with a global prevalence of 2-20%, and IBS has a significant impact on daily activities, school life, friendship and health related quality of life of affected children^2, 3)^. Therefore, the Rome IV criteria established diagnostic criteria for IBS in children and adolescents^4)^.

Colonic transit disorders may contribute to symptoms among patients with IBS; in fact, transit and contractile abnormalities have been observed in a subset of patients with IBS. The primary alteration of mucosal absorption and secretion has been suggested as non-contributory to the frequent stools with diarrhea in patients with IBS^5)^.

Several previous studies have shown a relationship between the accelerated transit of colonic contents with IBS and diarrhea. In animal models, previous studies have shown that stress loading increases stool frequency and causes diarrhea^6)^. Mönnikes et al. reported a significant acceleration of colonic transit in rats with restraint stress compared to that in non-restrained rats^7)^.

Furthermore, visceral sensation hypersensitivity from the autonomic nervous system associated with the brain-gut interaction is thought to be the pathophysiology of IBS^8)^. Corticotropin-releasing hormone (CRH) is an important factor in explaining the pathophysiology of brain-gut interactions. CRH stimulates the pituitary adrenocorticotropic hormone and increases intestinal motility in the human body^9)^. Recently, there has been a growing interest in serotonin (5-Hydroxytryptamine; 5-HT) because of its possible involvement in IBS. 5-HT released by enterochromaffin cells (EC cells) within the mucosa via intraluminal distension or irritation stimulates 5-HT3 receptors located on the primary afferent neurons of both splanchnic and vagal fibers, thereby modulating a sensory response^10)^.

Although increased stool water in diarrhea is thought to result from diminished contact time of the luminal contents with the colonic mucosa, it is well known that approximately 80% of ingested water is absorbed from the small intestinal mucosa^11)^. Therefore, small intestine dysfunction induces diarrhea in many diseases; however, the correlation between diarrhea and motility alteration in the small intestine among patients with IBS, especially childhood and adolescent age, has not been well evaluated. In this study, we aimed to assess the small intestinal transit in adolescent IBS rat models with restraint stress and to determine the role of small intestinal motility in the pathophysiology of IBS.

## Materials and Methods

### Animal models

The experiments were performed using adolescent male Sprague-Dawley rats aged 5-6 weeks as adolescent, weighing 160-250 g and were housed in cages in a standardized environment with a temperature of 24 °C, relative humidity of 55 % ± 15 %, and a 12-hour/12-hour light-dark cycle for a day. We chose male rats because we wanted to avoid female hormonal effects. They were allowed to access food and water freely. The animal care and experimental protocols were approved by the Institutional Review Board of Juntendo University (No. 310185).

The animals were randomly assigned to two groups: a restraint group and a control group. The restraint group was isolated in the individual compartments of stress cages (Natsume Seisakusho Co. Ltd. Tokyo, Japan; KN-325-C-3) for 1 hour before dissection. Rats in the control group assumed an hour of isolation in the cages without restriction. The rats in both groups did not have free access to food and water during isolation.

All methods were performed in accordance with relevant guidelines and regulations, and this study was conducted in compliance with the ARRIVE guidelines.

### Fecal pellet output and water contents

The number of fecal pellet outputs during the one-hour isolation was counted. The stool first excreted during isolation was collected, as well as the stool located in the most distal side of the gastrointestinal tract after isolation was collected for comparison. These fecal pellets were stored at a temperature of -80 °C and were freeze-dried overnight after weighing. The freeze-dried fecal weight was measured on the next day to calculate the water content (%), which was as follows: (fecal weight before drying - fecal weight after drying) / fecal weight before drying × 100 %.

### Small intestinal transit

**Figure 1:**
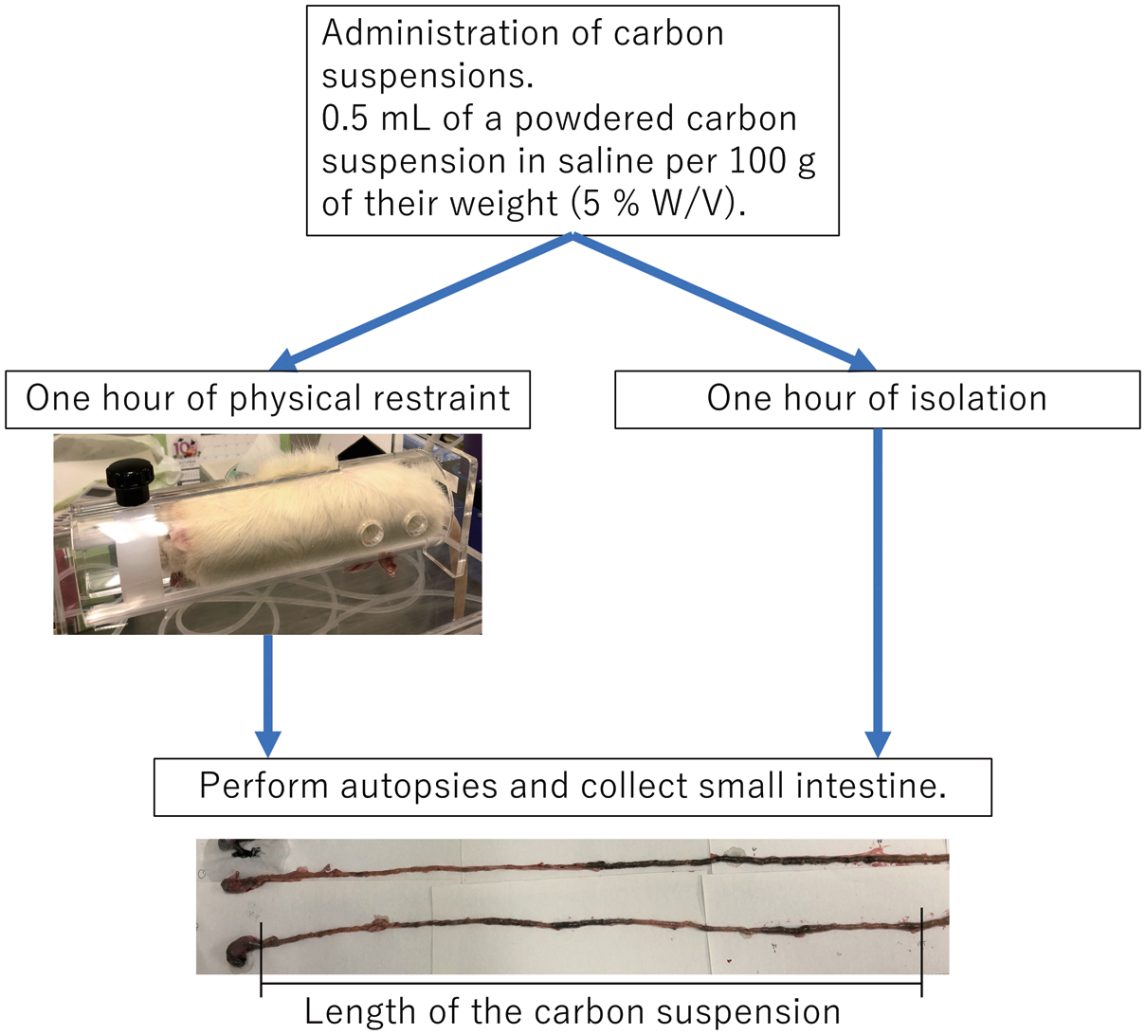
The process from administration of the carbon suspension to dissection was shown. After administration of the carbon suspension, the restraint group was restrained for one hour and the control group was isolated for one hour. After one hour of restraint and isolation, the animals were dissected. In this figure, I presented a picture of a rat in physical restraint. And also presented a picture of the removed small intestine. This picture was taken after the carbon suspension was administered. As shown, the area where the carbon had passed through was stained black, and this length was measured.

We examined the intestinal propulsion of powdered carbon to evaluate small intestinal motility under restraint stress. The rats received 0.5 mL of a powdered carbon suspension in saline per 100 g of their weight (5 % W/V) intragastrically through an oral sonde (Primetech Co. Ltd. Tokyo, Japan; FTP-15-78-50). After administration, the rats in the restraint group were immediately exposed to stress, as described above. Rats in both groups underwent an autopsy 1 hour after intragastric administration of the powdered carbon suspension. The process was shown in the figure (Figure 1). When the abdominal wall was opened during dissection, the intestinal tract was exposed. At that time, the intestines that had been exposed to carbon suspension were stained black and could be observed visually. The small intestines were collected, and their entire lengths placed naturally were measured. We also measured the length of the small intestine containing the carbon marker. The small intestinal transit rate (%) was calculated as follows: (the length of the small intestine containing the marker / total small intestinal length) × 100 %.

### Real-time polymerase chain reaction (PCR)

The amount of mRNA encoding CRH, mast cells, and 5-Hydroxytryptamine Receptor 3A (5-HTR3a) was quantified using real-time PCR. Full-thickness segments of the small intestine and proximal and distal colonic tissue samples were preserved in RNA stabilization solution and stored at -80 °C until use. Each tissue sample was homogenized in Tri Reagents (Tomy, Japan; MS100) to extract total RNA according to the manufacturer’s instructions (Applied Biosystems). Real-time PCR was performed using the 7500 Fast Real-Time PCR system (Applied Biosystems). The expression of each gene was normalized to the expression of glyceraldehyde 3-phosphate dehydrogenase (GAPDH) using the standard curve method. TaqMan was used for analysis using primers for CRH (Assay number Rn01462137_m1), mast cells (Assay number Rn04342812_g1), and 5-HTR3a (Assay number Rn00667026_m1). The results were compared between the restraint and control groups for both the small intestine and colon. Furthermore, the proximal and distal segments of the small intestine were also compared.

### Immunohistochemical analysis

Small intestine and colon tissues were dissected from rats and fixed in 4 % paraformaldehyde in 100-mM phosphate buffer at room temperature for 24 hours. Serial sections (4-μm thick) were prepared from formalin-fixed paraffin-embedded tissue sections of the small intestine and colon. For 5-HT detection, cells were incubated with 5HT (1: 10; Thermo Fisher Scientific, Rockford, USA) and then stained using the iVIEW™ DAB Detection Kit (Ventana) and Hematoxylin Counterstain II (Ventana). For 5-HTR3a detection, paraffin sections were heat-treated in Cell Conditioning Solution (Ventana Medical Systems) for 5-HTR3a, incubated with normal horse serum (Vector), rabbit anti-rat 5-HTR3a (Abcam), HRP-polymer-conjugated horse anti-rabbit IgG (Vector), and then stained using *ultra*View™ Universal DAB Detection Kit (Ventana) and Hematoxylin Counterstain II (Ventana). All the stains mentioned above were used according to the manufacturer’s protocol. An automated immunostainer (BenchMark; Ventana) was used to stain both 5-HT and 5-HTR3a. For each specimen, the number of 5-HT-positive cells was counted in six randomly selected fields per section using a KS400 Image Analyzer System (Zeiss). The data were expressed as the average number of positive cells per 400 µm2 of the mucosa.

Similar to real-time PCR, the restraint and control groups were compared for both the small intestine and colon; distal and proximal comparisons were also made in the small intestine.

### Statistical methods

All experimental data are presented as the mean ± standard deviation. All statistical analyses were performed using EZR^12)^ (Saitama Medical Center, Jichi Medical University, Saitama, Japan). More precisely, EZR is a modified version of R commander (version 2.7-0, 2021) designed to add statistical functions frequently used in biostatistics. As appropriate, the t-test was used for comparisons between the two groups. Differences between means at a level of *p* < 0.05 were defined as statistically significant.

### Data Accessibility

The data that support the findings of this study are available from Juntendo University, but restrictions apply to the availability of these data, as they were used under license for the current study and are not publicly available. However, data are available from the authors upon reasonable request and with the permission of Juntendo University.

## Results

### Fecal pellet output and water content

**Figure 2:**
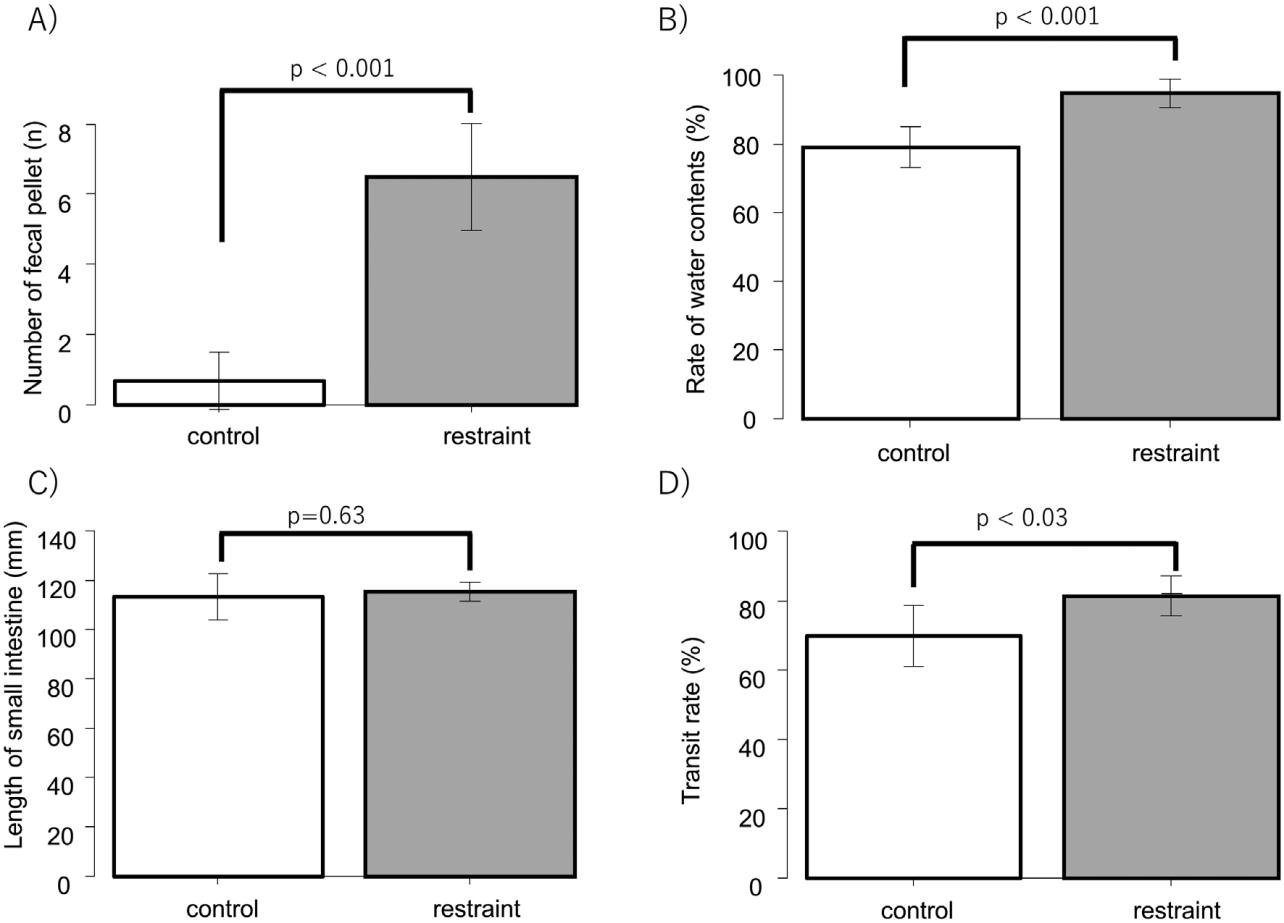
A: The number of fecal pellet outputs during 1 hour of isolation was counted. The restraint (n = 12) and control (n = 12) groups were compared. B: Comparison of the rate of fecal water content in the restraint (n=6) and control (n=6) groups. The first fecal pellet collected during isolation and after isolation was compared ((fecal weight before drying - fecal weight after drying) / fecal weight before drying (%)). C: The length of the small intestine was compared between the restraint (n = 9) and control (n = 16) groups. D: The transit rate of the small intestine was compared between the restraint (n = 9) and control (n = 16) groups. The small intestinal transit rate was calculated as the length of the small intestine containing the marker / total small intestinal length × 100%.

We counted the fecal pellet outputs of 12 restraint rats and 12 control rats. The mean number of fecal pellets was 6.9 ± 1.7 and 0.8 ± 0.8 in the restraint and control groups, respectively, showing that restraint stress significantly increased the number of fecal pellet outputs (*p* < 0.001, Figure 2A). We also calculated the water content of fecal pellets in six restraint rats and six control rats. The mean water content of the first stool during isolation was 75.5 % ± 7.5 % and 74.7 % ± 5.4 % in the restraint and control groups, respectively, with no significant difference in primary stools between the two groups during isolation (p = 0.80). On the other hand, the mean water content of the fecal pellets after isolation was significantly different between the restraint rats and control rats, at 94.8 % ± 4.2 % and 79.1 % ± 6.0 %, respectively (*p* < 0.001, Figure 2B). This result indicates that restraint stress led to increased water content in the stool and induced diarrhea in the stress models.

### Small intestinal transit

Nine restraint rats and 16 control rats were compared for small intestinal motility. The mean length of the small intestine was not significantly different between the restraint and control groups (115.3 ± 3.8 cm vs 113.3 ± 9.2 cm; *p* = 0.63) (Figure 2C). The mean small intestinal transit rate was 77.9 % ± 7.4 % in the restraint group and 69.5 % ± 8.9 % in the control group, with the restraint group showing a significant increase in the small intestinal transit rate (p < 0.03) (Figure 2D).

### Real-time PCR

**Figure 3:**
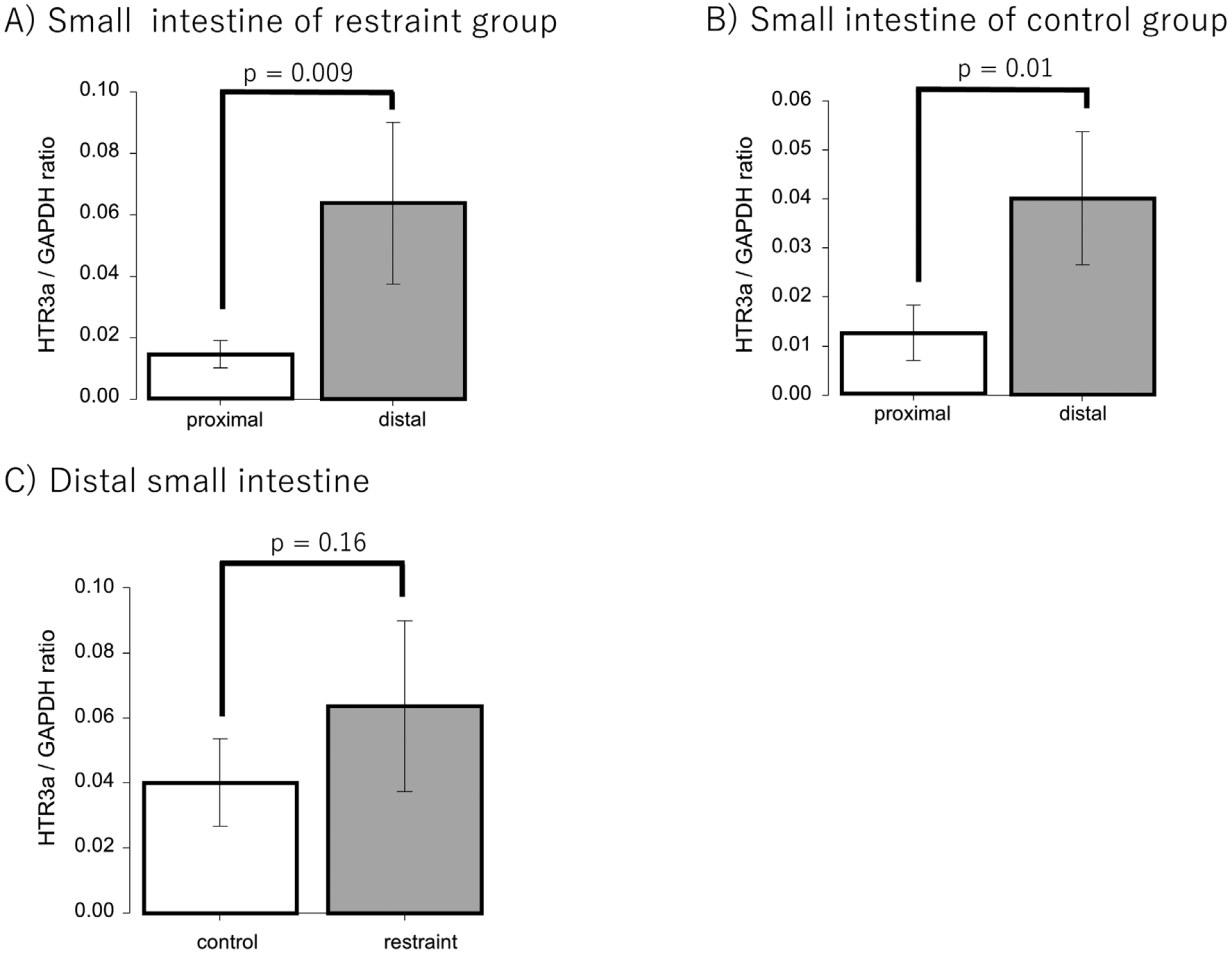
A: The expression of 5-Hydroxytryptamine Receptor 3A (5-HTR3a) in the proximal and distal small intestine was compared in the restraint group. B: The expression of 5-HTR3a in the proximal and distal small intestine was compared in the control group. C: The expression of 5-HTR3a was compared between the restraint and control groups only in the distal small intestine.

Six rats each in the restraint and control groups were compared for the expression of CRH, mast cells, and 5-HTR3a in the small intestine and colon using real-time PCR. The transcripts encoding these were normalized to those encoding GAPDH. We found that the mRNA expression was not significantly different between the restraint and control groups (Table 1). Examining the segment of the small intestine, 5-HTR3a expression was significantly increased at the distal portion compared to that at the proximal section in both the restraint and control groups (restraint: *p* = 0.009; control: *p* = 0.01) (Figure 3A, B). Moreover, in the distal small intestine, there was no significant difference in 5-HTR3a expression between the restraint and control groups (0.06 ± 0.03 vs. 0.04 ± 0.01: *p* = 0.16) (Figure 3C).

**Table 1:** Amount of mRNA encoding CRH, mast cell, and 5-HT receptor 3a mRNA expression in the small colon and colon as quantified by real-time PCR

		CRH	Mast cell	5-HTR3a
	Restraint group	－	1.50±1.50	0.03±0.02
Small intestine	Control group	－	2.36±2.42	0.04±0.03
	P value	－	0.408	0.336
	Restraint group	－	1.28±1.00	1.94±0.93
Colon	Control group	－	1.37±0.96	1.25±0.68
	P value	－	0.81	0.05

Values are mean ± standard deviation. There was no difference in CRH, 5-HT receptor and mast cell subunits between the restraint and control groups. CRH, corticotropin-releasing hormone; PCR, polymerase chain reaction; 5-HT, 5-Hydroxytryptamine; 5-HTR3a, 5-Hydroxytryptamine Receptor 3A

### Immunohistochemical analysis

**Figure 4:**
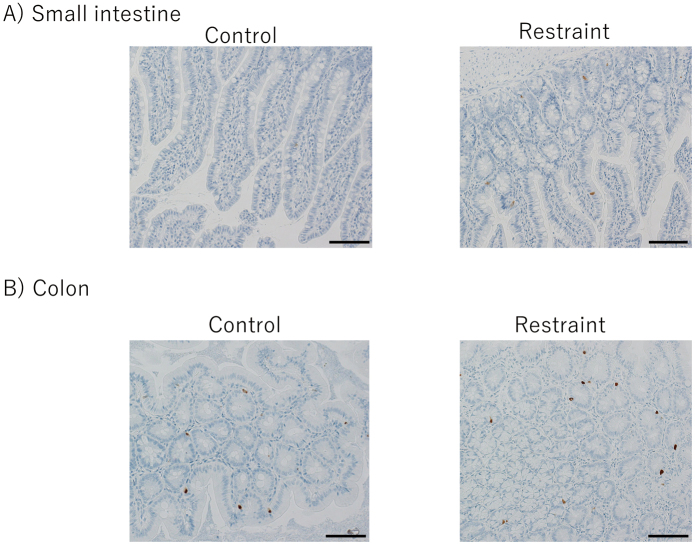
Enterochromaffin cell (EC cell) expression tended to increase in the restraint group compared to that in the control group in both the small intestine and colon. Bar = 100 μm. A: Photographs of the small intestine tissue. The positive cells (EC cells) were detected. The number of positive cells between the restraint and control groups was compared. B: Photographs of the colon tissue. The number of positive cells between the restraint and control groups was compared.

**Figure 5:**
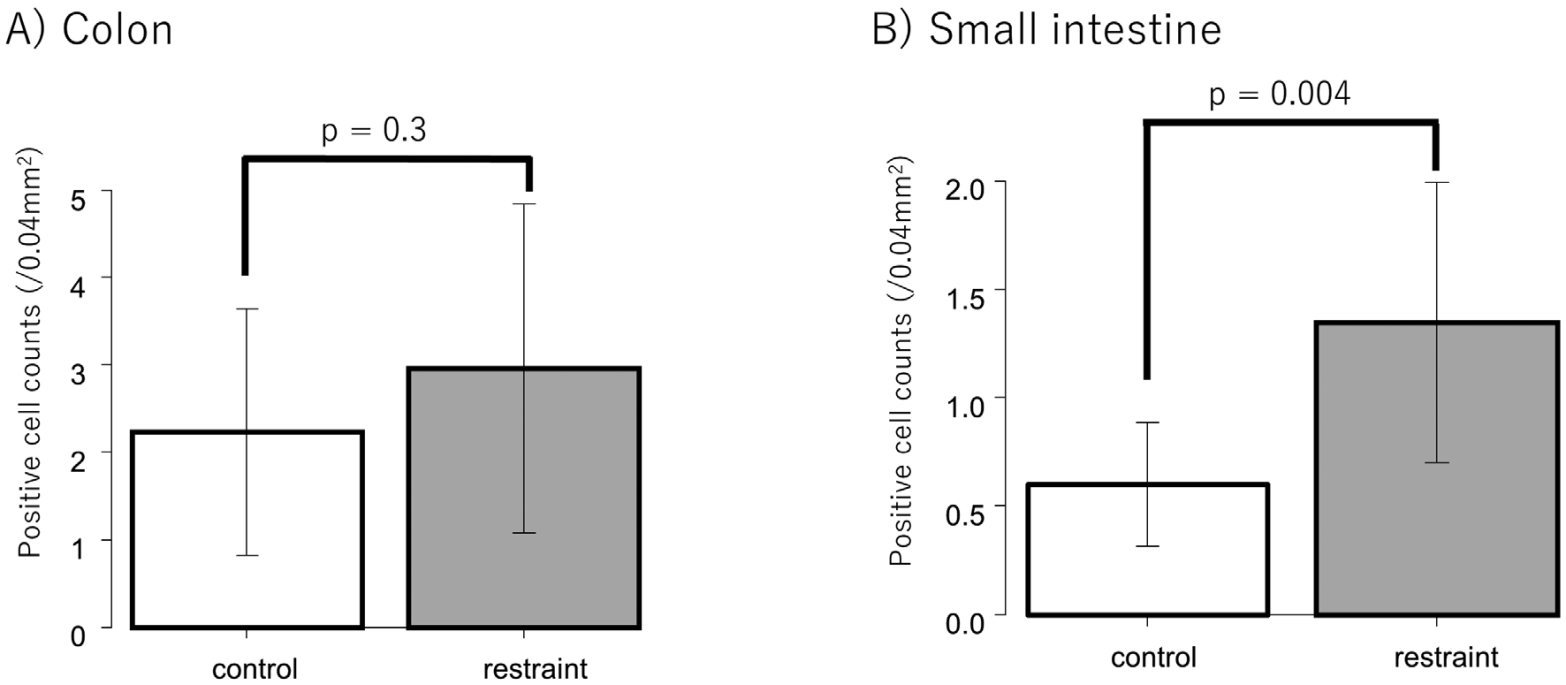
Comparison of the number of positive cells in the restraint (n = 6) and control (n = 6) groups and expression of 5-Hydroxytryptamine (5-HT) in the small intestine and colon. 5-HT is secreted from EC cells. EC cells that were stained and positive were counted. The number of EC cells in the small intestine increased in the restraint group, but not in the colon. A: The counted positive cells in the colon were compared between the restraint and control groups. B: The counted positive cells in the small intestine were compared between the restraint and control groups.

**Figure 6:**
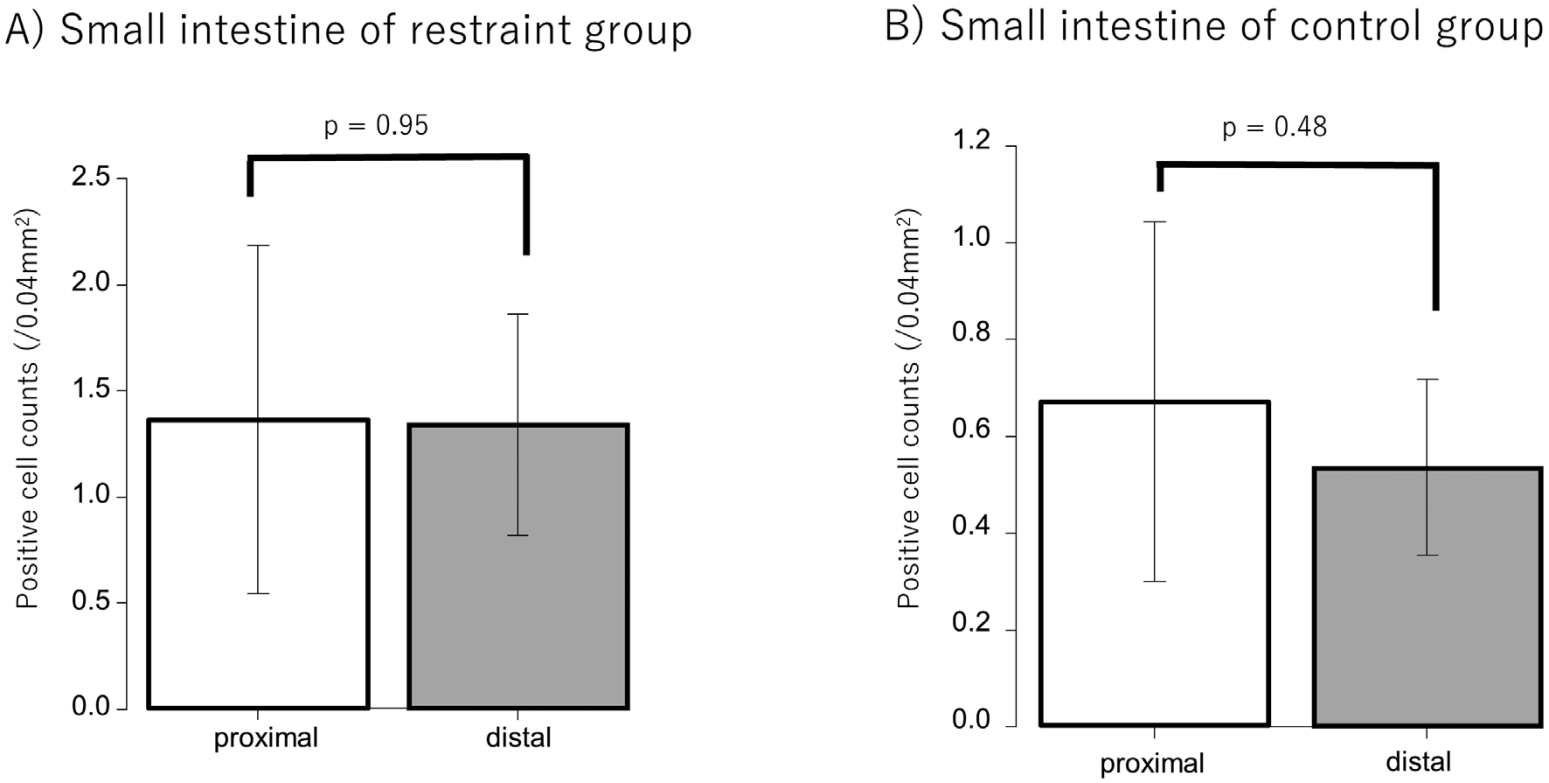
A: Comparison of enterochromaffin cell (EC cell) expression between the proximal small intestine and the distal small intestine in the restraint group. B: Comparison of EC cell expression between the proximal small intestine and the distal small intestine in the control group.

**Figure 7:**
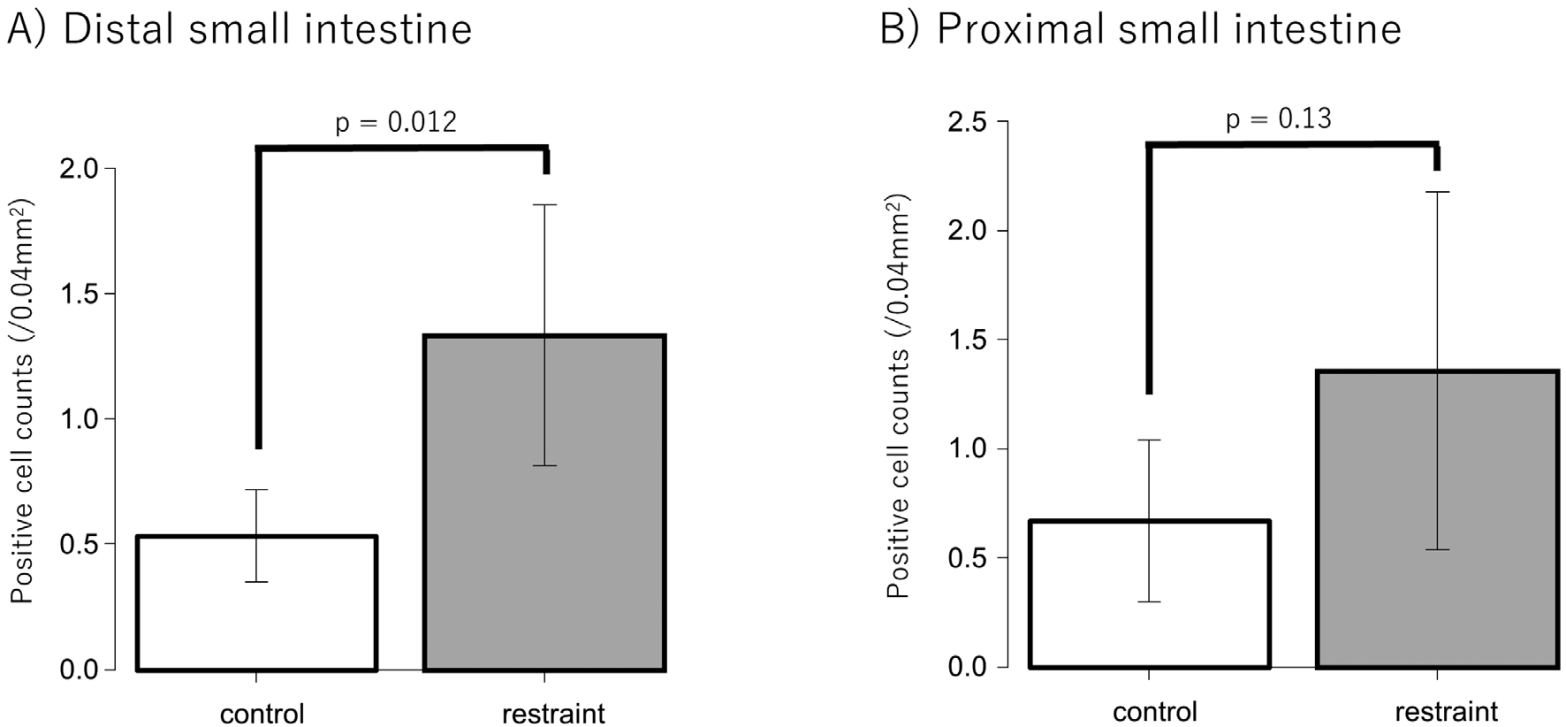
A: The number of Enterochromaffin cells (EC cells) in the distal small intestine was compared between the restraint and the control groups. B: The number of EC cells in the proximal small intestine was compared between the restraint and control groups.

**Figure 8:**
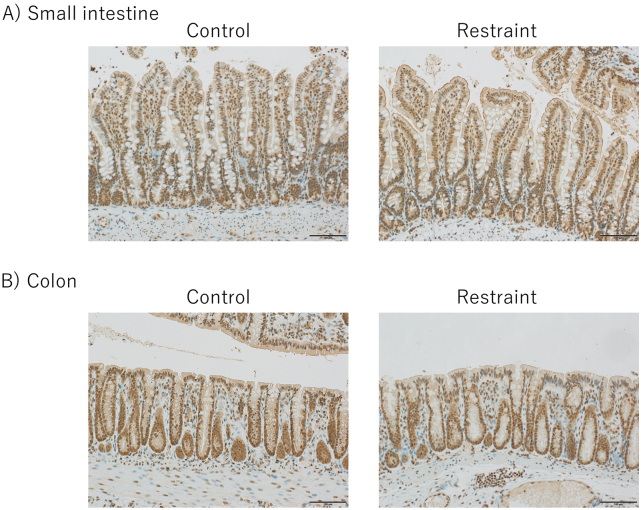
The expression of 5-Hydroxytryptamine Receptor 3A (5-HTR3a) in the small intestine and colon. Bar = 100 μm. A: Photographs of the small intestine tissue. The expression of 5-HTR3a between the restraint and control groups was compared. B: Photographs of the colon tissue. The expression of 5-HTR3a between the restraint and control groups was compared.

Immunostaining of 5-HT and 5-HTR3a was performed using tissues from the small intestine and colon. Six rats each from the restraint and control groups were used for comparison. Since 5-HT is secreted by EC cells, the 5-HT antibody staining of the small intestine and colon tissues was used to stain the EC cells. Positive cells in the control and restraint groups were counted and compared. As a result, EC cell expression tended to increase in the restraint group compared to that in the control group in both the small intestine and colon (Figure 4A, B). The number of positive cells was significantly higher in the restraint group than in the control group in the small intestine instead of the colon (small intestine: *p* = 0.004; colon: *p* = 0.3) (Figure 5). When the number of EC cells in the small intestine was compared between the proximal and distal small intestine, no significant difference was observed between the restraint and control groups (restraint group: *p* = 0.95; control group: *p* = 0.48) (Figure 6A, B). In the distal small intestine, the number of EC cells significantly increased in the restraint group compared to that in the control group (1.33 ± 0.52 vs. 0.53 ± 0.18, respectively; p = 0.012) (Figure 7A). In the proximal small intestine, there was no significant difference in the number of EC cells between the restraint and control groups (1.36 ± 0.82 vs. 0.67 ± 0.37, respectively; p = 0.13) (Figure 7B). Moreover, there was no significant difference in 5-HTR3a between the control and restraint groups in both the small intestine and colon (Figure 8).

## Discussion

Animal models of bowel motility dysfunction associated with stress induced by cold, acoustics, ether, cold restraint, and wrap restraint have been previously reported. For example, Murakami et al.^13)^ intravenously administered CRH to induce stress in rats. On the other hand, Bradesi et al.^14)^ used water-avoidance stress. Furthermore, restraint stress has been used in rat stress models. The number of defecations in rats reportedly increase by restraining the body^6)^. In the present study, adolescent rats subjected to restraint alone at room temperature showed an increase in the fecal pellet output without the formation of gastrointestinal lesions. We chose restraint stress to prepare IBS rat models because this stress method is simple and provides equivalent stress for each rat. In humans, IBS is diagnosed based on the clinical symptoms. Symptoms are very important to confirm IBS even in the animal models created in our experiment. In the present study, rats showed an increased fecal pellet output and diarrhea as clinical signs. Although we could not evaluate visceral hypersensitivity as no increased CRH release was observed, it was possible to create IBS rat models. There are three types of IBS: diarrheal, constipated, and mixed. The present study is a rat model of IBS with diarrhea.

Currently, most studies on IBS focus on visceral hypersensitivity and the brain-gut interaction. Therefore, it is important to understand gastrointestinal motility during IBS treatment. There are some reports about colon motility in IBS^15-17)^. However, Hardy et al.^18)^ reported that when comparisons were made between diarrhea-type and constipation-type IBS, it was observed that the colonic transit time in diarrhea-type IBS was shorter, whereas no difference in transit time was observed in the small intestine. Therefore, there was no influence on the small intestine movement in IBS. However, in the present study, small intestinal motility was significantly elevated, as observed in the small intestinal transit rate. Furthermore, colon motility was increased, as observed in the fecal pellet output and water content examination. Our IBS rat model had a shortened transit time in the entire intestine. In general, it is known that water is absorbed in the large intestine. However, in reality, 80% of the water we ingest is absorbed in the small intestine. Therefore, it is suggested that increased peristalsis of the small intestine may play a significant role in the appearance of diarrhea symptoms.

In this study, 5-HT, 5-HTR3a, and mast cells were evaluated in the colon and small intestine as factors that regulate the movement of the intestinal tract. Most 5-HT are synthesized and stored in EC cells, and 5-HT receptors are located throughout the intestinal tract. 5-HTR3 antagonists reportedly reduce visceral hypersensitivity and pain in patients with IBS^19)^. Therefore, 5-HT and 5-HTR3 play important roles in the gastrointestinal tract motility. However, their expression in the small intestine remains unclear. In our study, the motility of the small intestine in the restraint group as adolescent IBS model rats was significantly enhanced. Therefore, an increase in 5-HT and 5-HTR3 levels in the small intestine was expected. In the restraint group, EC cell expression in the small intestine was significantly increased, but not 5-HTR3a. This result suggests that 5-HT strongly affects the enhancement of small intestinal motility under acute stress. When the increase in EC cell expression in the small intestine among rats in the restraint group was evaluated with respect to the segment of the small intestine, we observed that the expression was significantly increased in the distal small intestine compared to that in the proximal small intestine. Furthermore, real-time PCR did not show a stress-related increase in 5-HTR3a in the small intestine; however, the distal small intestine expressed more 5-HTR3a than the proximal small intestine in both the restraint and control groups. Therefore, the distal small intestine may be mainly involved in small intestinal motility. The high expression of EC cells in the small intestine may result in increased intestinal motility and may be involved in developing diarrheal symptoms in IBS. This suggests that the small intestine is more susceptible to stress than the colon. In our study, real-time PCR showed no significant difference in mast cells between the restraint and control groups. However, there are some studies on an increased number of mast cells in patients with IBS. Chadwick et al.^20)^ reported that the number of mast cells increased in the colonic mucosa of patients with IBS. We found that 5HTR3a was not significantly increased by stress loading, but its expression was significantly higher in the distal small intestine. In addition, 5HT was increased by stress loading, but the increase was more prominent in the small intestine than in the colon. Furthermore, when compared within the small intestine, the increase in the distal small intestine was significantly greater than that in the proximal. Therefore, we believe that the increase of 5HT in the distal small intestine, where the expression of 5HTR3a is originally high, is strongly involved in the enhancement of small intestinal peristalsis. In the present study, we used stress loading, so the involvement of 5HT is likely to be large, but we believe that the site of high expression of 5HTR3a is also important. Weston et al.^21)^ reported that the number of these cells increased in the ileal mucosa of patients with IBS. It is possible that the reason the mast cells did not increase in our study was because the stress introduced was acute and not chronic. However, it remains unclear whether stress and IBS are correlated with an increase in the number of mast cells. The number of mast cells can also be increased by inflammation. Based on these facts, it can be reported that the adolescent IBS rat model created in our study had no inflammation and that the IBS rat model could be accurately created.

Nevertheless, our study had some limitations. First, in the creation of adolescent IBS model rats, the stress was acute, not chronic. While it might have been better if we chose chronic stress because IBS is a chronic syndrome, there was the possibility of ulcer formation due to chronic stress, which we wanted to avoid. We decided that chronic stress causing intestinal ulceration should not be a part of the evaluation, since this study dealt with functional intestinal disorders. Second, in the present study, only diarrhea-type IBS model rats were included. The pathogenesis of IBS includes diarrhea, constipation, and mixed types. Therefore, it is suggested that the results may differ depending on the pathology. Third, we were unable to evaluate increased CRH release. We tried to evaluate the increase in CRH using RT-PCR, but it could not be detected. Therefore, we determined whether the adolescent IBS rat model could be created based on clinical symptoms. Fourth, 5-HTR3a was evaluated using immunostaining; however, the difference in expression levels could not be quantified. RT-PCR did not show a significant increase in 5-HTR3a expression in both the small intestine and colon. If the expression level had been quantified using immunostaining, the evaluation would be more accurate. In the present study, the evaluation focused on 5-HT. However, there are other factors known to be involved in intestinal motility. We would like to evaluate these factors in future studies.

IBS model rats could be created by applying restraint stress. We found that the small intestine was prominently involved in enhancing intestinal peristalsis, which causes diarrhea. In particular, the distal small intestine of adolescent IBS model rats may be significantly involved in enhancing small intestine movement due to acute stress loading.

## Funding

The authors received no financial support for the research.

## Author contributions

T.K., M.S., T.I. and K.S. formulated the ideas, research goals and aims, and development or design of methodology; M.S., T.I., T.K. and Y.O. curated data and conducted formal analysis; N.A., R.K., K.H. and K.J. conducted the research and investigation process, specifically performing the experiments or collecting data/evidence; T.S. was entrusted with the leadership responsibility for the research activity planning and execution, including providing mentorship to the core team; M.S., T.K. and Y.O. created and/or presented the published work, specifically writing the initial draft; T.I. and T.K. performed critical review, commentary, or revision. All authors read and approved the final manuscript.

## Conflicts of interest statement

The Authors declares that there are no conflicts of interest.
